# Unmet Challenges in Patients with Crohn’s Disease

**DOI:** 10.3390/jcm12175595

**Published:** 2023-08-27

**Authors:** Katharina M Scheurlen, Mary A Parks, Anne Macleod, Susan Galandiuk

**Affiliations:** 1 Price Institute of Surgical Research, Hiram C. Polk Jr. MD Department of Surgery, University of Louisville, Louisville, KY 40202, USA; katharina.scheurlen@louisville.edu (K.M.S.); maryalex.parks@louisville.edu (M.A.P.); anne.macleod@louisville.edu (A.M.); 2 Division of Colon and Rectal Surgery, Hiram C. Polk Jr. MD Department of Surgery, University of Louisville, Louisville, KY 40202, USA

**Keywords:** Crohn’s disease, inflammatory bowel disease, tumor necrosis factor inhibitors, biomarkers, endoscopy

## Abstract

Patients with Crohn’s disease can present with a variety of clinical manifestations; treatment strategies should focus on long-term remission and improvement of quality of life. There is no standardized process of diagnosing, predicting prognosis, and treating the disease. This narrative review was based on a literature search using PubMed, Embase, and Science Direct. Data on unmet challenges in patients with Crohn’s disease were extracted from identified manuscripts. The aim was to discuss present research on standardized processes in the management of patients with Crohn’s disease and to identify the unmet needs in clinical evaluation and treatment approaches. There is no consensus on standardized diagnostic, treatment, and surveillance algorithms, particularly in assessing complications of Crohn’s, such as stricturing disease, intestinal cancer risk, and cutaneous manifestations. Complications and treatment failure rates of conventional, interventional, and surgical therapy place emphasis on the need for standardized treatment algorithms, particularly in the case of acute complications of the disease. Research on standardized clinical approaches, reliable biomarkers for disease diagnosis and therapy monitoring, and new treatment agents is necessary to improve therapy and reduce complications in patients with Crohn’s disease.

## 1. Introduction

Crohn’s disease can affect any part of the gastrointestinal tract, from mouth to anus. Depending on the severity of the disease and associated complications, such as fistula and strictures, symptoms vary among individuals with Crohn’s disease. This causes major difficulties in establishing a standardized process of diagnosing the disease. It is not uncommon for patients to have symptoms for years prior to diagnosis.

The severity of active inflammation poorly correlates with symptoms in patients with Crohn’s disease. Standardized approaches for diagnosis, treatment, and surveillance are lacking. This may facilitate under- or overtreatment, and places emphasis on serial evaluation of noninvasive biomarkers, as well as endoscopic assessment and cross-sectional imaging, to obtain an objective evaluation of disease behavior as a guide to therapy [1]. The goal of Crohn’s therapy is induction of remission of the disease and, due to its chronic course, maintenance of remission in the long term [2]. Treatment includes *5-*aminosalicylic acid** (5-ASA) and sulfasalazine compounds, corticosteroids, immunosuppressants, and monoclonal antibodies directed at immune cell trafficking and cytokine signaling [2,3]. Historically, corticosteroid treatment, immunosuppressive agents (such as thiopurines or methotrexate), and antibiotics have represented the keystones of conventional Crohn’s therapy [2]. During the last two decades, monoclonal antibodies have played an increasing role in the treatment of Crohn’s disease; however, therapy with tumor necrosis factor (TNF)-inhibitors is challenging due to high rates of nonresponse and secondary loss of response [3,4]. The long-term safety and effectiveness of newer biologicals, such as vedolizumab, ustekinumab, and risankizumab, have yet to be confirmed.

A consensus is particularly lacking for the management of common clinical scenarios in Crohn’s disease, such as the treatment of acute flares, of stricturing or cutaneous disease, or regarding cancer risk assessment.

The aim of this narrative review was to discuss the current research in the management of patients with Crohn’s disease, focusing on key unmet needs in clinical evaluation and treatment approaches, and to describe further research needs in Crohn’s disease.

## 2. Materials and Methods

This narrative review was supported with a literature search using PubMed, Embase, and Science Direct. Data from the current literature, up to February 2023, including clinical trials, prospective and retrospective observational studies, review articles, opinions, and guidelines, were reviewed. Data on unmet challenges in patients with Crohn’s disease were extracted from identified manuscripts. The keywords used for the literature search were “Crohn’s Disease”, “Outcome measures “, “Predictors”, “Biologic agents”, “Perianal Crohn’s disease”, “Surveillance”, “Stricturing Disease”, “Cancer risk”, and “Cutaneous Crohn’s Disease”. Following literature review and based also on the clinical experience of the senior author, we chose to focus on the following unmet needs:

- Accurate early disease diagnosis and phenotyping
- Variable disease course and response to treatment (including high treatment failure rates and lack of optimal disease monitoring, and decisions regarding the withdrawal of therapy)
- Standardized methods of surveillance
- Management of stricturing Crohn’s disease
- Management of cutaneous disease manifestations

A detailed outline of literature review with a PRISMA flowchart of search strategy and number of articles reviewed is found in Supplementary Figures S1–S4.

## 3. Results

### 3.1. The Unmet Need: Accurate Early Disease Diagnosis and Phenotyping

#### 3.1.1. What We Know

Although there are recent advances in the ability to accurately diagnose Crohn’s disease versus other subtypes of inflammatory bowel disease, there have been no major improvements in the overall time to diagnosis of Crohn’s disease, with the median time from symptom onset to diagnosis remaining at one year [5]. Most initial symptoms of Crohn’s disease, such as fatigue and abdominal pain, are vague, potentially subtle, and common to many other more benign, temporary pathologies [6]. Extraintestinal manifestations, which may be present in nearly a third of patients before the time of diagnosis, can further muddle the presentation. It is important to identify factors of initial patient presentation that could lead to a delay in diagnosis. Ajbar et al. (2022) describe several of these characteristics, including younger age of presentation, growth delay, and lack of diarrhea with initial presentation. Interestingly, they demonstrate that fistulizing disease is correlated with a longer time from symptom onset to diagnosis [7]. A systematic review by Jayasooriya et al. (2022) reported that a delay in diagnosis is associated with increased risk of penetrating or stricturing disease, at or following diagnosis. Delay was also associated with requirement for intestinal surgery, while no association was found with disease location [8].

**Figure 1:**
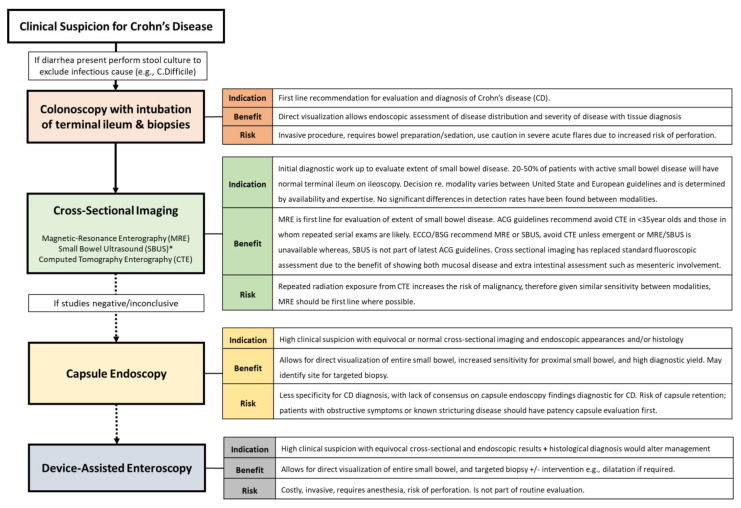
A diagnostic algorithm for Crohn’s disease. Imaging and endoscopic modalities algorithm for the diagnosis of Crohn’s disease with indication, risks, and benefits of each modality, created and adapted from most recent American College of Gastroenterology (ACG), European Crohn’s and Colitis Organization (ECCO), and British Society of Gastroenterology (BSG) guidelines [1,10,11]. * Guidelines were similar across most areas other than Small Bowel Ultrasound (SBUS) use—not part of ACG clinical guidelines. MRE: magnetic resonance enterography, CTE: computed topography enterography. CD: Crohn’s disease.

Although there is significant variability in disease presentation and initial severity, there are numerous non-invasive methods that can aid in the early diagnosis of Crohn’s disease. Fecal calprotectin measurement can be used to identify mucosal inflammation, and serum measures of systemic inflammation, including Erythrocyte Sedimentation Rate (ESR) and C-Reactive Protein (CRP), may aid in diagnosing an inflammatory or immune-mediated condition, although these laboratory measures are non-specific for Crohn’s disease [9]. Endoscopic assessment and cross-sectional imaging should be performed based on clinical suspicion of Crohn’s disease. A normal ileo-colonoscopy should not exclude Crohn’s disease (CD), as 30–50% of patients will have isolated small bowel disease. A diagnostic algorithm with indications, risks and benefits of each assessment modality is found in Figure 1.

#### 3.1.2. What Is Being Investigated

Recently, with the promotion of early intervention in Crohn’s disease, the idea of “preclinical inflammatory bowel disease” has gained popularity. This follows the principle of other immune-mediated diseases, in that there is a time before the onset of clinical symptoms during which immunological changes might be detectable in a patient’s serum [12]. Torres et al. (2020) demonstrated that a significant number of patients show elevated serum immunologic markers, such as anti-saccharomyces cerevisiae antibody (ASCA), perinuclear anti-neutrophil cytoplasmic antibodies (ANCA), and inflammatory markers, including interleukin-6 and CRP, even years before a diagnosis is made [12]. The main limitations for preclinical diagnosis are that there are no strict guidelines for interception of disease before presentation of symptoms, markers described are not Crohn’s disease specific, and, at present, a clear, high-risk population to screen has not been identified [13].

Early diagnosis of Crohn’s disease remains an important and potentially life-altering unmet challenge for these patients. There is also often an incorrect categorization or phenotyping of Crohn’s disease. While edematous inflammatory disease will readily respond to medical therapy, dense fibrosis will not. Early diagnosis allows for prompt, aggressive management to control inflammation prior to the onset of symptomatic complications of chronic inflammation. A systematic review by Steiner et al. (2022) identified 35 distinct biomarkers that have been studied in the diagnosis of fibrosis in Crohn’s disease. These included serum (*n* = 20), histopathological (*n* = 6), and genetic (*n* = 9) markers. In 15/18 studies, mutations in the NOD2/CARD gene were found to be strongly associated with the presence of sticturing CD; however, mutations were not specific to stricturing disease. Similar results were noted for serum ASCA positivity, which was sensitive, but not specific, for stricturing disease [14]. Presently, neither European (ECCO) nor American (ACG) guidelines recommend the routine use of serological biomarkers or genetic analysis in the diagnosis of Crohn’s disease.

**Figure 2:**
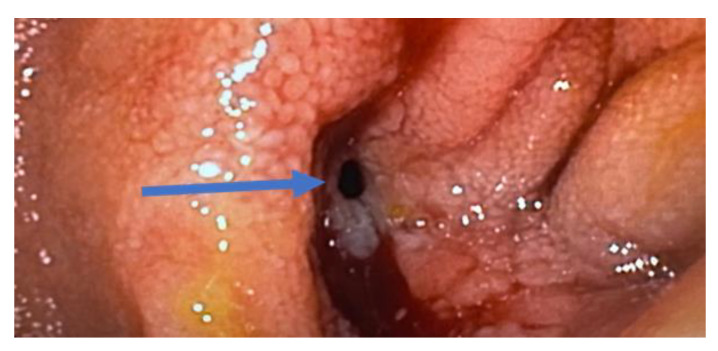
Endoscopic view of a small bowel fistula due to Crohn’s (arrow).

Ulcers, strictures, and fistulas develop as a result of uncontrolled mucosal inflammation (Figure 2). The goal of early initiation of biologic therapy is to prevent the development of these architectural changes associated with long-standing disease that may ultimately require surgery. Increasing knowledge and awareness in the medical community can potentially lead to increased detection and initiation of treatment earlier in the course of the disease before such significant structural changes in the bowel, e. g., strictures and fistulas, occur.

#### 3.1.3. Remaining Gaps/Future Directions

Current studies into diagnostic biomarkers both for early detection and correct phenotyping are largely limited by their retrospective nature and associated confounding factors. Prospective studies into biomarkers, such as antimicrobial antibodies and serum peptides with identified cut off values, along with genetic variants in Crohn’s disease to predict stricturing disease, are warranted. A multi-center trial (NCT04088773) is currently recruiting patients undergoing ileal resection for stricturing small bowel disease to evaluate serum and stool biomarkers, to correlate with magnetic resonance imaging (MRI) findings, and to compare these with those of patients with non-fibrostenotic Crohn’s and those of healthy controls.

Future work is also required to identify specific, high-risk populations and the potential benefits of screening. Crohn’s disease is a multifaceted disease with complex genetic, environmental immune-related risk factors; at present, there is insufficient evidence for the use of screening in specific high-risk groups based on presence of risk factors alone.

### 3.2. Variable Disease Course and Response to Treatment (Including High Treatment Failure Rates and Lack of Optimal Disease Monitoring, and Decision Regarding Withdrawal of Therapy)

#### 3.2.1. The Unmet Need: Variable Disease Course and Response to Treatment

##### What We Know

There are many presentations of Crohn’s disease, along with a spectrum of effects on quality of life. Many of the complications of Crohn’s disease, such as fibrotic strictures, abscesses, and colorectal cancer can severely limit quality of life. Current medications, including biologics, can effectively manage disease and prevent many complications [15]. In the setting of inflammatory bowel disease, the term “biologic” refers to monoclonal antibodies, administered either intravenously or subcutaneously to induce and maintain disease remission. Unfortunately, the decision to start biologic therapy is not as easy as one might imagine. The high cost of such therapy and the possible side-effects are reasons for caution that are cited by both patients and physicians [16]. However, there is increasing evidence to suggest that early biologic use improves rates of clinical remission and reduces the rate of penetrating complications in pediatric patients [17]. Ultimately, the inability to accurately predict disease outcomes in Crohn’s disease in a way that could direct therapy represents a substantial unmet need in these patients.

##### What Is Being Investigated

There are proposed prognostic tools used to predict long-term disease course in Crohn’s disease, such as the pediatric RISK stratification study model used as an indicator to predict which patients will develop complications requiring surgery [17] and the PROSPECT study model that uses serologic and genetic testing, disease phenotype, and demographics to predict the risk of a complication of disease occurring within three years [16]. Both tools have significant limitations, such as poor generalizability to all Crohn’s disease patients [16]. PredictSURE is a blood test that measures the gene expression of CD8+ T cells, to aid in predicting the likely course of disease for newly diagnosed Crohn’s disease, including a patient’s likelihood to be hospitalized or require surgery for their condition [18]. This test has yet to be studied in the United States [18].

##### Remaining Gaps/Future Directions

Further investigation is needed to determine diagnostic predictors of long-term outcomes of Crohn’s disease. Initial disease presentation may be misleading, as some individuals present with acute flares that respond to treatment, while others experience moderate or refractory disease, with some eventually leading to complications or requiring surgery [19]. It is imperative to determine reliable tools for classifying patients who will likely develop a severe disease course and who are therefore reasonable candidates for aggressive biologic, or perhaps earlier surgical, therapy.

#### 3.2.2. The Unmet Need: High Treatment Failure Rates and Optimal Disease Monitoring

##### What We Know

Treatment of Crohn’s disease is aimed at successful induction and maintenance of remission, thereby reducing incidence of complications and, overall, improving a patient’s quality of life. Ultimately, the use of biologics has changed the natural history of Crohn’s disease for many patients, preventing disease progression rather than simply controlling clinical symptoms. Specifically, infliximab and adalimumab as monotherapy are both able to induce clinical and biochemical remission even in cases of severe Crohn’s disease [20].

Despite the ability of these pharmacologic interventions to improve outcomes in a subset of patients, it is estimated that up to one-third of patients will not respond to anti-TNF therapy. Additionally, there is a second subset of patients who may initially respond to these treatments but lose response over time or become intolerant to these medications. These numbers may reach as high as 30–40% of patients during the first year of anti-TNF therapy [21]. Overstraeten et al. describe the inadequacies of biological therapy, stating that the rate of small bowel resection after attempted treatment with biologics has not decreased. Complications of Crohn’s disease, including the frequency of stricturing, along with hospitalization trends, have not substantially improved with the use of biologics [22]. Overall, it is estimated that eighty percent of these patients will eventually require surgery at some point [23]. Because of the high treatment failure rates with current biologics ultimately leading patients to continue to experience disease progression and complications, it is imperative to investigate further pharmacologic and non-pharmacologic treatment options, including novel biologic therapies with varying mechanisms, diet interventions, fecal transplantation, and others.

##### What Is Being Investigated

The STRIDE (Selecting Therapeutic Targets in Inflammatory Bowel Disease) initiative highlights the importance of early introduction and alteration of mediation according to well defined goals as the crucial basis of managing CD [24]. They categorize clinical/symptomatic response as an immediate treatment target, clinical remission as intermediate, and endoscopic healing as a long-term target. The trials discussed below (Table 1) use these endpoints in reporting and evaluating the success of new drug targets for CD. A normalized, health-related quality of life is an important long-term target and is increasingly seen as a secondary endpoint in randomized clinical trials (RCTs) evaluating new IBD treatments.

The “prototype biologics” infliximab and adalimumab are TNF-inhibitors, essentially blocking the production of proinflammatory cytokines during the inflammatory response, but this mechanism is non-specific [25]. This has led to the investigation of other methods of altering the immune response in Crohn’s disease, such as anti-adhesion molecules, janus kinase (JAK) inhibitors, and anti-IL-23 agents [26,27]. Anti-adhesion therapies work to limit the interaction of leukocytes and endothelial cells, effectively preventing the migration of leukocytes to the intestinal mucosa [28]. Ustekinumab is a biologic that has been approved for the treatment of Crohn’s disease since 2016, which targets interleukin-23 and interleukin-12, which are both involved in the T-helper pathways thought to have a role in the pathogenesis of Crohn’s disease; however, similar to TNF-inhibitors, there has been a demonstrated loss of response to ustekinumab in about one-third of patients [29]. Additionally, there is thought to be a beneficial role of interleukin-12 in the protection of tumor-promoting properties; by blocking IL-12, ustekinumab theoretically has the potential to increase the risk of malignancy [30]. This led to the development of four monoclonal antibodies that specifically target interleukin-23 alone [31]. Another mechanism currently under exploration for targeting the immune response in Crohn’s disease involves targeting the downstream effects of inflammatory cytokines by inhibiting the intracellular JAK-signal transducer [32].

**Table 1:** Current biologics and new drugs recently approved/under investigation for the management of Crohn’s disease.

Current Biologics Recommended in Crohn’s Disease			
Biologic Family Examples	Mechanism of Action	Secondary Failure Rates/ Complications	Guideline Recommendation *
**Anti-TNFα** -Infliximab -Adalimumab -Certolizumab -Golimumab	Non-specific inhibition of pro-inflammatory cytokines via TNF blocking.	20–30% non-responders 30–40% initial responders, secondary failure.	Induction of remission and maintenance+/- thiopurine/methotrexate for maintenance. Patients should undergo latent/active tuberculosis testing prior to commencing.
**Anti-IL12/23** -Ustekinumab	Targets p40 subunit on IL12/23 part of the T-helper cell pathway.	Similar failure rates to anti-TNFa (30–50%) Risk of pro-tumorigenic effect of blocking IL-12—new biologics targeting IL-23 alone are in development.	Induction of remission and maintenance or loss of response to anti-TNF therapy.
**Anti-integrins** -Natalizumab Anti-α4 -Vedolizumab Anti-α4β7	Targets leukocyte trafficking via cell-adhesion molecule (CAM). **Natalizumab** acts via Vascular CAM-1 and Mucosal Addressin CAM-1 **Vedolizumab** is gut-specific via Mucosal Addressin CAM-1 only.	Risk of PML (progressive multi-focal leuko-encephalopathy) with **Natalizumab** if John Cunningham (JC) virus antibody positive. **Vedolizumab** is gut specific with no CNS uptake, no reported cases of PML. Failure rates in 34.3% monotherapy and 29.3% combination with thiopurine [33].	Induction of remission and maintenance or loss of response to anti-TNF therapy. **Natalizumab** should not be used in combination with immuno-modulators. John Cunningham (JC) Virus serum antibody testing prior to commencing and every 6 months. AGA (2021) guidelines suggest against the use of Natalizumab [34]. **Vedolizumab** can be used in combination with immunomodulators.
**New Drugs Recently Approved/Under Investigation for use in Crohn’s Disease**			
**Drug Family ** Mechanism of Action	**Drug Name**	**Completed Trials/Current Evidence**	**Active/Ongoing Trials.**
**Anti-IL23** Targets p19 subunit of IL-23. A promotor of Th17 cell immune response [31].	**Risankizumab** Approved for use in Crohn’s disease (June 2022).	Phase 3 RCTs **ADVANCE** and **MOTIVATE** found Risankizumab was well tolerated and effective for the induction of remission (45% and 42%) vs. placebo in moderate to severe CD with loss of response to previous biologics [35]. Follow-up maintenance **FORTIFY** trial found greater clinical (52% vs. 41%) and endoscopic (47% vs. 22%) response vs. placebo [36].	Phase 3 **SEQUENCE** trial comparing Risankizumab vs. Ustekinumab. NCT04524611 [37]
	**Mirikizumab**	Phase 2 **SERENITY** trial, in patients with ileocolic or colonic CD, favorable endoscopic response (43.8% vs. 10.9%) and clinical remission (20.3% vs. 1.6%) in the highest dose treatment group vs. placebo. Maintenance remission at 52 weeks was 50–63.3% [38].	Phase 3 trials **VIVID-1** (NCT03926130) [39] and long term extension **VIVID-2**, (NCT04232553) [40].
	**Brazikumab** (MEDI2070)	Phase 2 trial showed favorable safety profile in patients with moderate to severe Crohn’s who had failed anti-TNF. Clinical response at week 8 was found in 49.2% vs. 26.7% placebo with sustained response at week 24 [41]. IL-22 serum levels were found to be predictive of response to Brazikumab. Potential target for selecting appropriate patients for this treatment.	Phase 2b/3 **INTREPID** trial: Brazikumab vs. adalimumab vs. placebo NCT03759288 [42]. Phase 3 open label extension trial **INTREPID-OLE** studying long term safety NCT03961815 [43].
	**Guselkumab**	Phase 2 **GALAX-1** trial, showed efficacy in clinical response (60–64%) and remission (50–58%) in all doses of Guselkumab at 12 weeks. Results were similar to those treated with ustekinumab, although not a primary endpoint of the study [44].	Phase 3 **GRAVITI** Guselkumab efficacy and safety NCT05197049 [45]. Phase 3 **FUZION-CD** Guselkumab in peri-anal CD NCT0534709 [46]. Phase 2 trial **DUET CD** Combination with Golimumab (anti-TNF) NCT05242471 [47].
**Anti-TL1A** Tumor necrosis factor like ligand 1A. Blocks TL1A- involved in T cell activation, Th2 pathological responses, and production of IL-5 and IL-3 in intestinal mucosa.	PRA023	Phase 2a **APOLLO-CD** proof of concept trial reported 26% endoscopic response and 49% clinical remission at 12 weeks. Efficacy preserved in patients with previous biologic exposure [48].	Placebo-controlled trials awaited to confirm findings.
	TEV-48574		Phase 2b **RELIEVE UCCD** efficacy and dose response for remission using TEV-48574 NCT05499130 [49]. Phase 2b **RELIEVE UCCD LTE,** long term extension of **RELIEVE UCCD.** Efficacy and dose response for maintenance regimens NCT05668013 [50].
**JAK-Inhibitors** Inhibit JAK/TYK pathways, which mediate signal transduction downstream of many cytokines.	**Filgotinib** JAK1 inhibitor	Phase 2 **FITZROY** trial of 174 patients with ileal, ileocolonic or colonic CD. Induction of clinical remission was reported in 47% vs. 23% placebo following 10 weeks treatment [32]. Phase 2 **DIVERGENCE-**1 trial of filgotinib vs. placebo in Small Bowel Crohn’s disease showed no significant Difference in clinical remission, or MRI index of activity.	Phase 3 **DIVERSITY** NCT 02914561 [51] completed, awaiting publication of results. Phase 3 **DIVERSITY LTE** Long Term Extension NCT02914600 [52]. Long term efficacy and safety of filgotinib vs. placebo for remission induction and maintenance
	**Upadacitinib ** JAK-1 inhibitor. Approved May 2023 for moderate to severe CD with intolerance to or inadequate response from anti-TNF.	Phase 2 **CELEST** trial of efficacy and safety of multiple doses of Upadacitinib vs. placebo. Upadacitinib was superior in the induction of clinical and endoscopic remission as well as remission maintenance at 36 weeks compared to placebo [53]. Phase 3 **U-EXCEL** and **U-EXCEED** trials showed clinical remission in 39–50% vs. 21–29% in placebo, and endoscopic response 35–46% vs. 4–13% placebo. **U-ENDURE** maintenance trial reported clinical remission at 52 weeks in 37–47% patients vs. 15% placebo [54].	Observational **UPLIFT** trial to assess speed of onset and durability of effectiveness of Upadacitinib NCT05930275 [55]. Not yet recruiting.
	**Tofacitinib** (-approved for use in UC) Non-selective JAK inhibitors.	Two Phase 2 randomized, double blinded, placebo-controlled trials involving 460 patients showed no benefit over placebo with Tofacitinib for induction or maintenance of remission in CD [56].	No new/active trials at present.
	**Deucravacitinib** (BMS-986165) Tyrosine Kinase 2 (TYK2) inhibitor.		Phase 2 **LATTICE-CD** trials for long term efficacy and safety of Deucravacitinib. NCT03599622, NCT04877990 [57]
**Sphingosine-1-** **Phosphate** **Receptor** **Modulators** Inhibit leukocytes uptake in peripheral tissues.	**Ozanimod** S1P1, S1P5 selective.	Phase 2 **STEPSTONE** trial reported endoscopic response in 23.2% and clinical remission in 39.1% of patients [58].	Phase 3 **YELLOWSTONE** trial program including 2 placebo-controlled induction trials (NCT03440372, NCT0344038) 1 maintenance (NCT03464097) and an open label extension study (NCT03467958) [59].
	**Etrasimod** S1PR1, S1PR4, S1PR5 selective.	Phase 2 **CULTIVATE** trial Sub-study A suggested endoscopic and clinical improvement following etrasimod, but small numbers and no placebo limit conclusions [60].	Phase 2/3 **CULTIVATE** trial includes 5 sub-studies evaluating efficacy, safety, and tolerability of etrasimod. NCT04173273 [61]

* Guideline recommendation based on summary of most recent American College of Gastroenterology (ACG), American Gastroenterological Association (AGA), European Crohn’s and Colitis Organization (ECCO), and British Society of Gastroenterology (BSG) guidelines [1,2,11,34]. TNF: Tumor Necrosis Factor, IL: interleukin, JAK: Janus Kinase, TYK: Tyrosine Kinase.

##### Remaining Gaps/Future Direction

Proactive drug-level testing is a potential approach to monitor maintenance therapy and prevent acute flares, but current evidence is lacking [46]. Levels of biologic drugs can be measured to determine biologic failure [1]. A major limitation is the uncertainty of optimal drug levels for many biologic therapies, particularly for newer ones. The most recent American Gastroenterological Association (AGA) guideline on Therapeutic Drug Monitoring in Inflammatory Bowel Disease suggests target trough levels for patients on maintenance therapy with TNF-alpha inhibitors (infliximab ≥ 5 μg/mL, adalimumab ≥ 7.5 μg/mL, and certolizumab pegol ≥ 20 μg/mL) [46]. The impact of proactive drug-level testing on the prevention of flares remains to be determined. In addition, the cost of such testing is expensive and not covered by many insurance plans in the US.

Future work is required into the augmentation of treatment regimens with other nonpharmacologic approaches. Preliminary studies on fecal microbiota transplantation have shown efficacy in the maintenance of clinical remission along with an overall improvement in clinical, endoscopic, and histological status [62]. An important advantage of this therapy is the overall limited number of adverse events reported in these preliminary studies compared to many pharmacologic interventions; however, more randomized-control trials are needed to fully assess the efficacy and safety of fecal transplantation [62]. Lastly, diet has been an important consideration as an adjunctive treatment for Crohn’s disease. Preliminary studies have found that the Crohn’s disease exclusion diet alone, a diet focused on whole foods and limiting exposure that could negatively affect the gut microbiome, has the potential to induce remission in mild-to-moderate biologic naïve patients [63]. Randomized control trials are necessary to establish efficacy versus other management strategies.

- Treatment Dilemma—Medical Treatment Versus Early Surgery

Debate continues regarding the optimal timing, and order, of surgery vs. escalation of medical therapy in patients with active Crohn’s disease. Advances in medical therapy have led to the perception that avoidance of surgery is the goal of Crohn’s disease management. While often true, in patients not responding to initial immunomodulator therapy, escalation to biologics may not always be superior to surgery.

The multi-center LIR!C trial compared laparoscopic ileocolic resection vs. infliximab in patients with isolated, ileocecal non-stricturing, immunomodulator resistant Crohn’s disease [64,65]. Initial quality-of-life outcomes were equivocal between groups. At 5 years follow-up of patients who went straight to surgery, 26% required subsequent anti-TNF therapy; however, 29 (42%) required no further Crohn’s disease related medication [64,65]. No patients required further surgery. In the Infliximab arm, 48% of patients required a subsequent Crohn’s disease related resection, and the remaining 54% continued on a biologic [64,65].

Given the limitations and failure rates of biologic therapy as discussed above, continued trials and escalation of medical therapy, if unsuccessful, increases the risks associated with subsequent surgery. The systemic effects and complications associated with prolonged, active, uncontrolled disease, including disease progression, infection, malnutrition, and anemia, increase the peri- and post-operative risks of bowel resection, as well as the likelihood of requiring a stoma. Patients in this situation often also require courses of steroids, adding further peri-operative risk.

While advances in therapeutic options continue, identifying predictors or markers of responsiveness is crucial and remains an unmet challenge in tailoring patient-specific treatment. Phenotypic and genetic differences likely exist between patients with responsive and non-responsive disease. Early identification of those markers and predicting patients unlikely to respond to escalating medical therapy affords those patients the best chance at successful surgery with the least additional risk.

- Treatment Dilemma—When to Stop Biologic Agents

In the case of a successful treatment with biologics, young Crohn’s patients in remission, in particular, are exposed to multiple risks due to long-term maintenance on anti-TNF therapy. Apart from cost implications, an increase in cancer risk has been discussed, such as lymphoma, skin cancer, or HPV-associated malignancies [66]. There are no recommendations on the duration of treatment following induction of remission, and hard evidence on the associated cancer risk is lacking [66,67,68]. Recent studies, however, concluded that anti-TNF therapy and newer biologics may not increase the risk of new-onset or recurrent cancers in patients with inflammatory bowel disease [69,70].

### 3.3. The Unmet Need: Standardized Methods of Surveillance

#### 3.3.1. Surveillance for Recurrent Disease

##### What We know

It is well-known that symptomatology and mucosal inflammation correlate poorly in Crohn’s disease. As such, it is critical to define effective methods of surveilling disease activity, including the use of biomarkers, imaging, and therapeutic drug monitoring. The phase III CALM trial aimed to compare treatment regimens of endoscopic and biomarker management versus clinical management alone. The primary endpoint was mucosal healing with absence of deep tissue ulcers 48 weeks into the trial. It was demonstrated that tight control using symptoms, biomarkers, and endoscopy was significantly more effective in inducing remission than management driven by symptoms alone [71]. Further-more, symptom-based scoring systems, such as the Crohn’s Disease Activity Index (CDAI) and the Harvey–Bradshaw Index, have several limitations in surveillance scoring, such as room for variation in question interpretation, response subjectivity, and poor correlation overall with biomarkers [72].

Patients with Crohn’s disease should undergo surveillance (i) within 12 weeks of initiation of treatment for evaluation of clinical response, (ii) at 6 months for endoscopic or transmural assessment of response to therapy, (iii) regularly (every 3–6 months) during periods of asymptomatic disease, (iv) during a suspected new flare, and (v) within 6 months–1 year following ileocolic resection. Recommendations of timing intervals and modalities for this vary across society guidelines and reflect the heterogeneity of the disease course of Crohn’s disease between patients. There is a lack of evidence available to construct a uniform surveillance algorithm. In general, ileo-colonoscopy is the gold standard for surveillance. Non-invasive fecal and serological measures are important non-invasive first line investigations (discussed below). Cross-sectional imaging is a useful adjunct in symptomatic disease or to assess for response to therapy, although a reliable reference standard for disease activity is lacking. All new flares should have infective causes excluded prior to further investigation. The goal of surveillance following resection or during asymptomatic periods is to identify and treat early mucosal recurrence in order to avoid developing clinical recurrence.

Video Capsule Endoscopy (VCE) can be used in surveillance as well as diagnosis (Figure 2). It is particularly beneficial in patients with proximal small bowel disease and a normal ileo-colonoscopy. Clinical trials use quantitative measures of activity, such as the Lewis Score or Capsule Endoscopy Crohn’s Disease Activity Index (CECDAI). Capsule endoscopy is a well-tolerated and less invasive modality, but it is susceptible to over-interpretation of non-significant lesions. The risk of capsule retention is low; however, patients with previous resection, stricturing disease, or obstructive symptoms should undergo a patency capsule evaluation first [1,10,73].

##### What Is Being Investigated

Endoscopic healing after medical therapy in Crohn’s disease is associated with an overall better disease outcome. Perhaps one of the most important uses of endoscopy for Crohn’s disease monitoring is its predictive value for risk of surgery, risk of hospitalization, and even risk of postoperative recurrence and relapse [74,75,76]. Interestingly, patients with partial mucosal healing versus complete mucosal healing did not have a higher rate of surgery, emphasizing the need to determine a degree of mucosal healing required to determine disease outcome [74]. Additionally, it remains unclear whether treatment based on endoscopic surveillance or reported symptoms is more effective. This will be further evaluated in the randomized control trial REACT 2 [77].

Although endoscopy is the current standard method of disease assessment, its use is limited by routine availability, cost, and rare but potential complications [44]. As such, it is important to define non-invasive measures of disease activity for regular surveillance, such as the use of biomarkers, specifically CRP, and fecal calprotectin. CRP is produced by the liver in response to inflammatory cytokines and can theoretically be used to assess the level of inflammation at a given time point. In fact, it is suggested that CRP is a fairly sensitive biomarker for Crohn’s disease [78]. The low specificity of this biomarker is, however, a major limitation when considering its use in monitoring changes in mucosal inflammation. Not only can CRP levels increase in any inflammatory condition, but it is also estimated that as many as 20% of healthy individuals do not express CRP under conditions of inflammation [77]. Fecal biomarkers, such as calprotectin, have the potential to detect gut inflammation with higher specificity than CRP. Calprotectin is a calcium and zinc binding protein, and it is released into the intestine during leukocyte trafficking. Analyses have demonstrated that fecal calprotectin has a 93% sensitivity and 96% specificity, specifically for differentiating inflammatory bowel disease from non-organic causes of similar symptoms [77]. Overall, fecal calprotectin is of greater value in diagnostics and monitoring because of its greater sensitivity for inflammation [77].

##### Remaining Gaps/Future Directions

An additional aspect to consider when monitoring patients with Crohn’s disease is the potential for future treatment failure secondary to immunogenicity or poor pharmacokinetics of biologic agents with certain patients. Evidence suggests that maintenance of optimal drug concentration through therapeutic drug monitoring may allow for improved clinical outcomes [79]. As discussed earlier, early intervention in Crohn’s disease has become increasingly encouraged, and, as such, it should be pushed in order to optimize initial treatment response through monitoring for immunogenicity, or through the idea of neutralizing antidrug antibodies causing the acceleration of drug clearance, along with measurement of patient factors, such as body size and serum albumin concentration, to account for variable pharmacokinetic properties between patients [79].

Despite the known benefit of tight monitoring of Crohn’s disease using biomarkers, imaging, and therapeutic drug monitoring, there are several unmet needs when implementing this idea into practice, such as determination of which monitoring tools to use, the frequency with which to use them, and how different patient presentations warrant different monitoring strategies [80]. Additionally, objective, reproducible scoring systems for clinical practice are necessary, with accurate and reliable endoscopic cut-offs for treatment escalation and validated biomarkers of disease activity [80].

#### 3.3.2. Surveillance for Cancer

##### What We Know

Cancer risk is increased in patients with Crohn’s disease due to inflammatory stress and immunosuppressive medication [81,82,83]. The reported relative risk in patients with Crohn’s disease is increased by 30 percent for small bowel adenocarcinoma and by 3 percent for colon cancer in case of colonic Crohn’s activity [84,85]. Therefore, initial screening colonoscopy with surveillance biopsies is recommended beginning at year 8 after diagnosis and subsequently every 1–5 years, depending on family history and disease extent [86]. Evidence of the association between Crohn’s strictures and colorectal carcinoma is lacking. Retrospective data on the risk of colorectal cancer and stricturing disease are inconclusive. While some retrospective data suggest that colonic strictures are not independently associated with colorectal cancer, other data showed that patients with stricturing disease have a 5% 10-year risk [87,88]. In case of long-term stricturing disease, an underlying malignancy should be contemplated, and screening with biopsies should be considered. An elevated risk of colorectal cancer, however, has been reported even after short disease duration [88]. Dysplasia or cancer as an incidental finding after stricture resection have been diagnosed in 2% and 1% of patients, respectively [89].

##### What Is Being Investigated

Evidence is lacking on the benefits of routinely performed endoscopic biopsy of Crohn’s strictures. Routine biopsies remain controversial. Screening for dysplasia and malignancy in strictures is indicated, but it is made more difficult by the inability to obtain adequate biopsies due to the fibrotic nature of the tissue. Furthermore, malignant lesions may be missed, as biopsies are usually too superficial [90].

##### Remaining Gaps/Future Directions

Prospective studies investigating colonic Crohn’s are necessary to determine the role of cancer screening in colonic strictures. Malignant small bowel strictures are challenging to diagnose and are uncommon. Future studies should focus on diagnostic testing guidelines to detect small bowel malignancy at an early stage and to prevent delayed diagnosis in patients with long standing stricturing disease.

### 3.4. The Unmet Need: Management of Stricturing Crohn’s Disease

#### 3.4.1. What We Know

**Figure 3:**
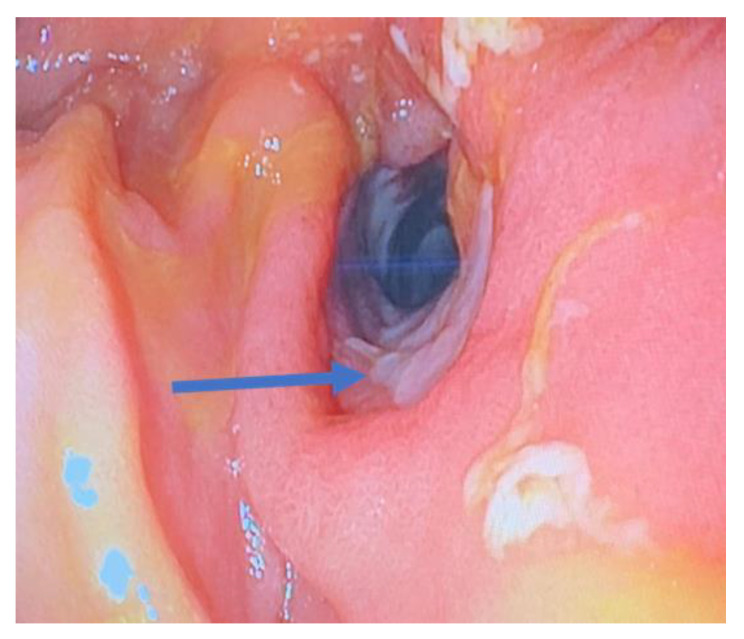
Endoscopic view of small bowel Crohn’s stricture with associated ulceration (arrow).

Stricturing disease in patients with Crohn’s disease can occur in both the upper and lower gastrointestinal tract (Figure 3). The most common site for fibro-stenosing disease is the terminal ileum and the ileocecal valve [90]. Chronic inflammatory stress with an abnormal response to wound healing causes transmural fibrosis. The amount of fibrosis increases during the course of the disease [81]. Medical therapy can reduce inflammation, but does not reverse fibrosis, and endoscopic dilation of a stricture is more likely to be successful the less scar tissue is present. Diagnostic imaging including computerized tomography (CT), magnetic resonance imaging (MRI), positron emission tomography (PET), and ultrasound are highly sensitive and specific in detecting a stricture. None of these techniques, however, provides reliable information on the proportion of fibrotic tissue, inflammatory infiltrates, and muscular hypertrophy within a stenotic bowel segment [91].

#### 3.4.2. What Is Being Investigated

Currently, there is not enough evidence to provide a general treatment algorithm for stricturing Crohn’s disease. In general, nonsurgical management of symptomatic Crohn’s strictures is preferred. If medical and endoscopic treatments fail and acute bowel obstruction develops, surgery is indicated.

- Medical therapy

Conservative therapy is the preferred first treatment approach in Crohn’s patients with symptomatic strictures in the absence of an acute bowel obstruction. The impact of specific anti-inflammatory medication on strictures in patients with Crohn’s disease, however, has not been systematically investigated in high-quality clinical trials [81]. Medical treatment is more effective in newer inflammatory, less fibrotic strictures. Corticosteroids can decrease inflammation and are commonly used in the acute setting. Anti-inflammatory drugs reduce inflammation over time to prevent fibrosis and have been shown to improve symptoms in stricturing Crohn’s disease [92]. A multicenter retrospective study showed that early treatment initiation within 18 months of Crohn’s diagnosis is associated with higher effectiveness of anti-TNF therapy (infliximab and adalimumab) [92]. Another retrospective study reported good short-term and modest long-term treatment response without the requirement for steroids, but with only 30% of patients retaining treatment due to lack of response, cost, and adverse events (infliximab and adalimumab) [93].

The prospective cohort study on contrast-enhanced ultrasound and MRE to predict the efficacy of anti-TNF therapy (CREOLE) from 2018 demonstrated that adalimumab treatment was successful in two-thirds of patients with stricturing disease; however, half of the included patients still required surgery within four years of follow-up [94]. The recently published randomized controlled Stricture Definition and Treatment (STRIDENT) trial showed that, apart from symptom improvement in approximately 70% of patients, adalimumab induced stricture resolution by reducing the inflammatory morphology of strictures in a significant proportion of patients [95].

- Endoscopic dilation

Endoscopic evaluation can help characterize a stricture, according to Paine and Shen, by describing its location, etiology (primary versus anastomotic), shape, and degree of obstruction, and by identifying associated lesions, such as ulcers, fistula, or malignancy [96]. The opportunity to perform interventions during endoscopic evaluation, such as balloon dilation of strictures, makes endoscopy a primary therapeutic option before surgical resection is necessary. Although endoscopic dilation is commonly performed, the heterogeneity of studies evaluating this technique allow no clear conclusions regarding long-term outcomes. Clinical efficacy, adverse events, and surgical intervention rates vary among studies, and the majority of investigated strictures are anastomotic and short in nature [97,98].

Endoscopic reintervention was necessary in up to 74% of patients, and up to 43% required surgical resection during highly variable follow-up periods [97,98]. There is no consensus on the required balloon inflation time for stricture dilation, but 1–3 min are generally performed [91,99]. A maximum dilation reaching 16–18 mm in diameter has been shown to be associated with longer intervals between subsequent dilations [100]. Strictures of up to 4–5 cm can be dilated, while longer strictures show an increased risk for surgery after endoscopic dilation [101].

- Surgical therapy

Patients that poorly respond to medical and endoscopic therapy require surgery. To identify patients requiring future surgery, Stidham et al. developed a tool considering small bowel dilation >35 mm on imaging (CT or MRI) and the platelet:albumin ratio > 125 as predictors for necessary surgical intervention within two years [102].

Operative complications are increased in patients with a poor nutritional status, perioperative steroid use, and open surgery [91]. Malnourished patients should receive perioperative nutritional support, either enteral or parenteral, and steroids should be tapered before elective surgery [103]. Laparoscopic approaches show similar success rates compared to open surgery, but are associated with shorter recovery and hospitalization periods, reduced rates of bowel obstruction, and better cosmesis [104]. Ileo-colonoscopy should be performed within one year following surgery to predict postoperative outcome and to evaluate for the presence of inflammatory activity, anastomotic strictures, and other complications, along with endoscopic classification according to the Rutgeerts score [105].

Surgical therapy consists of resection of the strictured bowel segment and/or strictureplasty and is not curative. Following bowel segment resection, recurrent anastomotic stricturing can occur in up 40% of patients within ten years after the initial operation [106]. Starting or re-establishing immunosuppressive therapy within four weeks postoperatively is associated with longer disease-free intervals in adults [107]. Rates of recurrent surgery following resection of fibrostenotic disease decreased significantly after biologics use was established.

**Figure 4:**
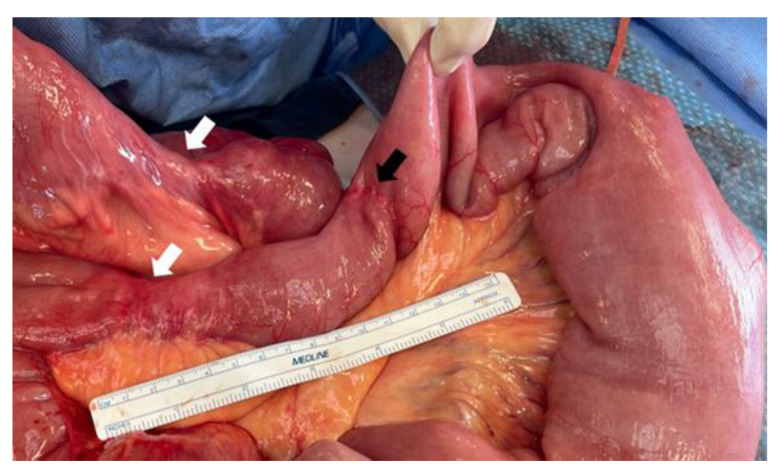
Patient with stricturing jejunal Crohn’s disease. Note massive dilatation of jejunum proximal to short fibrotic strictures (white arrows). These occurred years after a segmental resection proximally, which shows no evidence of recurrent disease (black arrow).

Strictureplasty may be indicated in cases of multiple shorter fibrotic strictures in the absence of infection, such as phlegmon, abscess, fistula, or perforation [108]. This technique preserves bowel length and can prevent short bowel complications in patients with a history of prior bowel resection. For strictures shorter than 10 cm in length, the Heineke–Mikulicz method is a common strictureplasty technique, while the Finney strictureplasty is suggested for longer strictures up to 25 cm in length [108]. Postoperative complication rates of strictureplasty are similar to those of bowel resection. Bowel resection for stricturing Crohn’s disease, however, was reported to be associated with longer recurrence-free survival [82]. In patients with stricturing small bowel Crohn’s disease, one of the unmet needs is prevention of new stricture development in other areas of the small bowel, as re-stricture of strictureplasty sites is rare (Figure 4).

#### 3.4.3. Remaining Gaps/Future Directions

- Medical therapy

Further trials are needed to investigate the long-term effectiveness of different biologics in larger patient cohorts with stricturing disease.

- Surgical therapy

The choice of anastomosis can have a significant impact on postoperative outcome after bowel resection—stapled intestinal anastomoses, both side-to-side and end-to-end, show superior outcomes compared to hand-sewn techniques (2% vs. 14% leak rate) [91,108]. Promising results have been reported concerning a newer technique, the Kono-S anastomosis. This technique protects the anastomosis from the adjacent small bowel mesentery. In order to obtain comparable data on leak rates and other complications of the Kono-S anastomosis, however, high-quality studies are still required, and multiple prospective randomized controlled trials are ongoing [108].

### 3.5. The Unmet Need: Management of Cutaneous Crohn’s Disease

#### 3.5.1. What We Know

Crohn’s disease is a systemic disease that may present with cutaneous manifestations in up to 43% of cases [83]. Cutaneous manifestation can be divided into five different categories, depending on the pathogenetic and histopathological findings of the respective lesion (Table 2) [84,85].

#### 3.5.2. What Is Being Investigated

**Figure 5:**
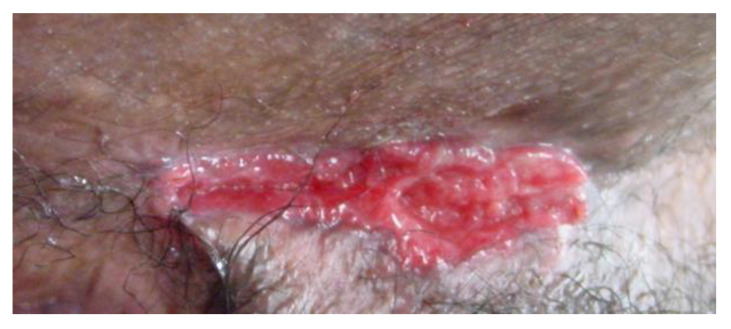
Cutaneous Crohn’s in an inguinal crease.

Cutaneous Crohn’s disease, the actual dermatologic manifestation of Crohn’s, de-scribed as category 1, is common, and treatment guidelines are lacking. Continuous or contiguous disease is part of this category and occurs in the perianal region as perianal erythema, abscesses, and/or complex fistulas [84]. The rare scenario of cutaneous Crohn’s as part of category 1, representing the only manifestation of the disease even in the absence of gastrointestinal tract involvement, is termed “metastatic Crohn’s” [86]. Metastatic Crohn’s disease, also called noncontiguous cutaneous Crohn’s disease, is defined as Crohn’s-specific non-caseating cutaneous lesions at distant sites from the gastrointestinal tract [84]. Lesions can include erythematous plaques, nodules, abscesses, fistulas, hidradenitis suppurativa-like lesions, and/or ulcers, most often found on the lower limbs and in intertriginous areas (Figure 5) [84,87].

#### 3.5.3. Remaining Gaps/Future Direction

Topical or systemic corticosteroids, azathioprine, methotrexate, or anti-inflammatory treatment with TNF-inhibitors is often not effective, and evidence on the effects of newer biologics on cutaneous Crohn’s disease is limited [87]. TNF-inhibitors and older biologic agents, such as infliximab or adalimumab, are often associated with paradoxical skin reactions (category 4) [89]. The newer biologic agent ustekimumab, however, has been reported to induce treatment response of cutaneous Crohn’s, which suggests a role of this biologic agent as a first-line agent in these cases [88,89]. Furthermore, ustekimumab seems to be effective in the treatment of drug-induced paradoxical skin reactions [89]. Further studies are needed to determine the efficacy of newer biologics and to develop a treatment algorithm for these.

## 4. Conclusions

The management of Crohn’s disease should follow a multidisciplinary approach and remains challenging due to the heterogeneity of clinical manifestations and individual treatment responses in patients. There is no consensus on standardized diagnostic, treatment, and surveillance algorithms, particularly with respect to complications of Crohn’s disease, such as acute flares, stricturing disease, intestinal cancer risk, and cutaneous manifestations. Widely used standard scoring systems to diagnose and classify Crohn’s disease have not yet been established. High conservative treatment failure rates encourage ongoing research on new anti-inflammatory and immunosuppressive targets and strategies. Complications and treatment failure rates of interventional and surgical therapy in patients with Crohn’s disease place emphasis on the need for standardized treatment algorithms, particularly in the case of acute complications of the disease. Research on standardized clinical approaches, reliable biomarkers, new treatment agents, and the treatment of the variety of clinical manifestations is necessary to improve therapy and reduce complications in Crohn’s disease patients.

## Figures and Tables

**Table 2:** Categories of cutaneous manifestations in Crohn’s disease. Modified after Antonelli et al. and Ungureanu et al. [84,85].

Cutaneous Manifestation Category	Definition
1	Disease-specific lesions. Same histopathological findings as Crohn’s. - Continuous/contiguous disease - Metastatic Crohn’s disease
2	Mucocutaneous lesions associated with Crohn’s.
3	Reactive (inflammatory) lesions that share a pathogenetic mechanism with Crohn’s disease, but not the same histopathology.
4	Drug-related mucocutaneous lesions secondary to Crohn’s treatment.
5	Cutaneous lesions secondary to nutritional absorption.

## Data Availability

No new data were created or analyzed in this study. Data sharing is not applicable to this article.

## Supplementary Materials

The following supporting information can be downloaded at: https://www.mdpi.com/article/10.3390/jcm12175595/s1, Figure S1: PRISMA Flowchart of literature review and search strategy for studies reviewed for Unmet Need 1: Accurate Early Disease Diagnosis and Phenotyping. Figure S2: PRISMA Flowchart of literature review and search strategy for studies reviewed for Unmet Need 2&3: Variable disease course and response to treatment & standardized methods of surveillance. Figure S3: PRISMA Flowchart of literature review and search strategy for studies reviewed for Unmet Need 4: Management of stricturing Crohn’s disease. Figure S4: PRISMA Flowchart of literature review and search strategy for studies reviewed for Unmet Need 5: Management of cutaneous disease manifestations.
