# Supplementary material for: Unmet Challenges in Patients with Crohn’s Disease
Source: J Clin Med. 2023 Aug 27;12(17):5595. doi: 10.3390/jcm12175595 (PMC10488639; doi:10.3390/jcm12175595)
Supplement: Supplementary file 1 [file jcm-12-05595-s001.zip › jcm-2405189-supplementary.pdf]

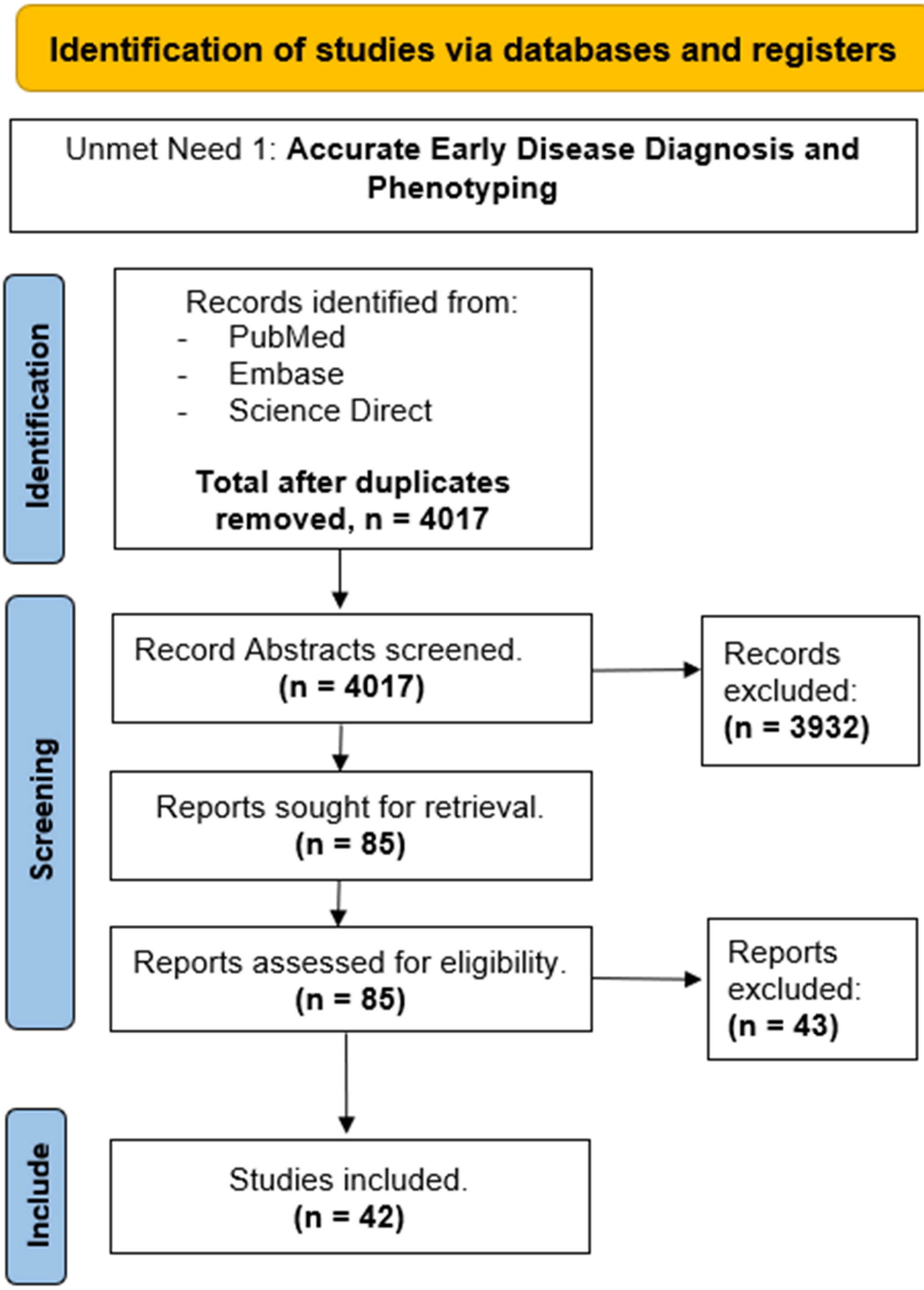


**Supplementary Figure S1:** PRISMA Flowchart of literature review and search strategy for studies reviewed for **Unmet Need 1: Accurate Early Disease Diagnosis and Phenotyping.** Search Terms: *((Crohn's Disease) AND (Presentation) AND (Time to Diagnosis) OR (Preclinical Inflammatory Bowel Disease Markers) OR (Biomarkers in Inflammatory Bowel Disease)* Results included studies from 2013-2023.

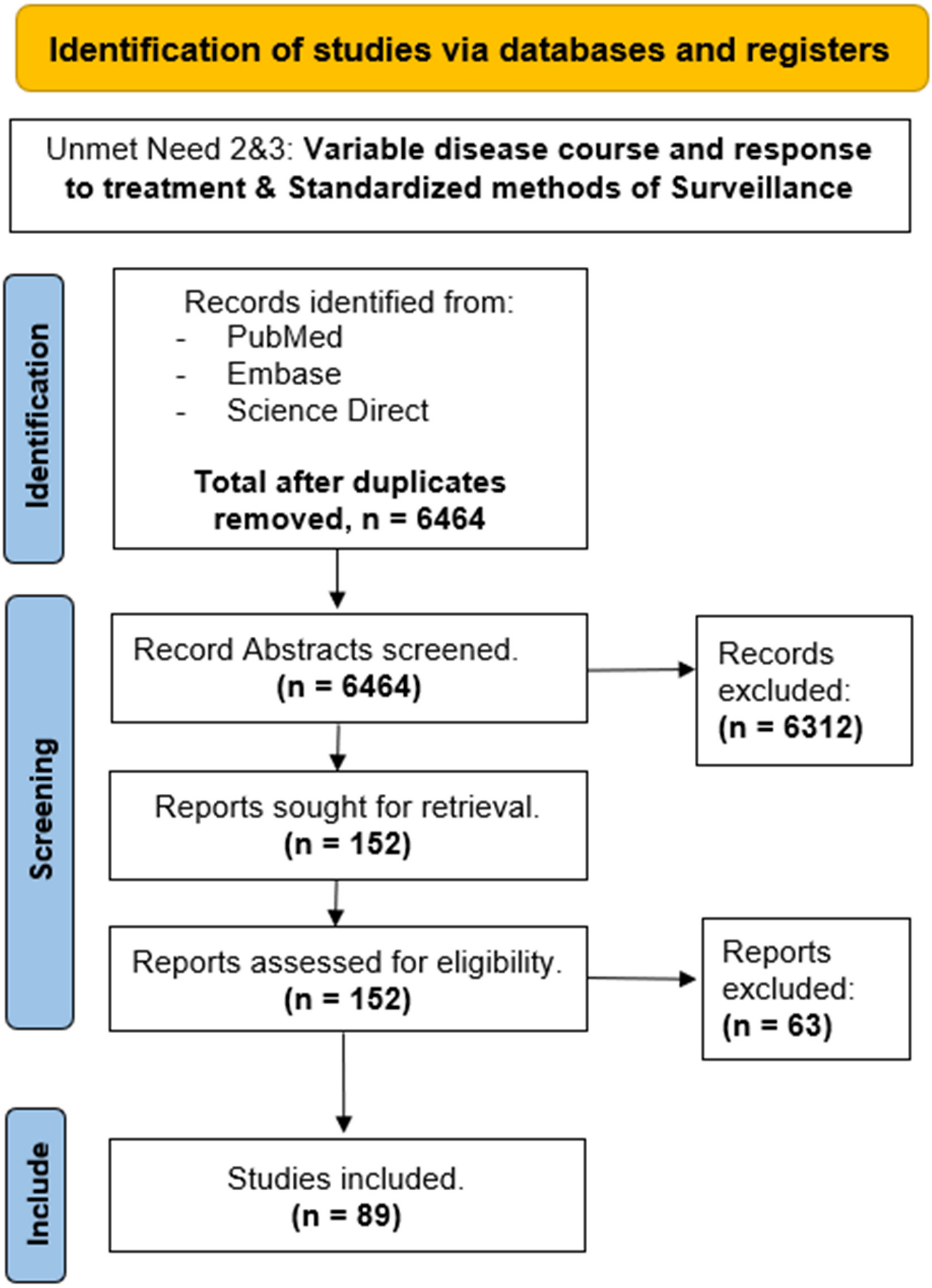


**Supplementary Figure S2:** PRISMA Flowchart of literature review and search strategy for studies reviewed for **Unmet Need 2&3: Variable disease course and response to treatment & standardized methods of surveillance.** Search Terms: *((((((Crohn's Disease[Mesh Terms]) AND (Biologic Efficacy)) OR (Crohn's Disease[Mesh Terms] AND Disease Monitoring) OR (Crohn's Disease [Mesh Terms] AND non-Pharmacological Management)) OR ((Crohn's Disease[Mesh Terms]) AND (Prognostic Tools)) OR (Crohn's Disease [Mesh Terms] AND Response To Treatment) OR (((Crohn Disease[Mesh Terms]) AND (Cancer Surveillance)))* Results included studies from 2013-2023.

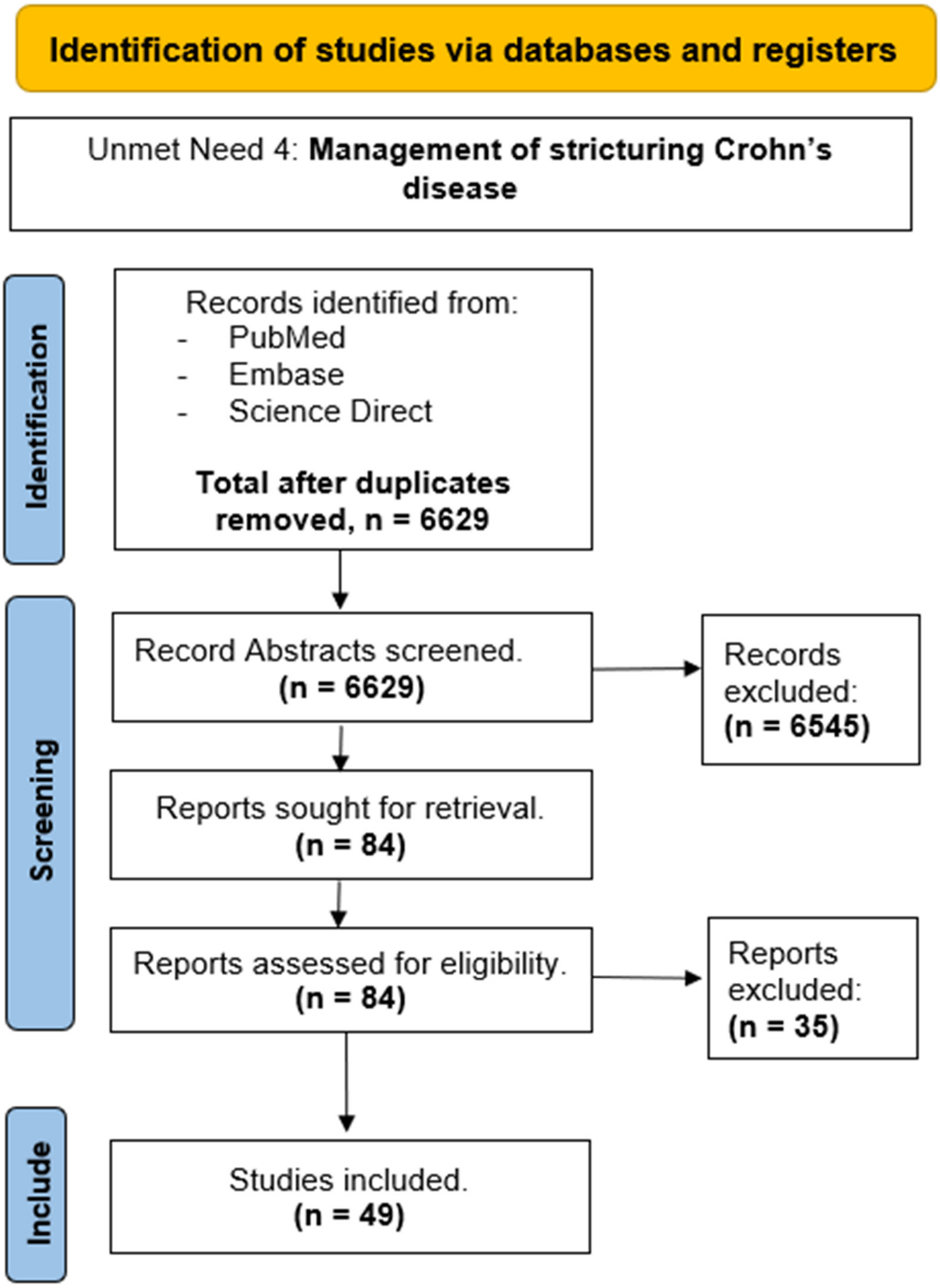


**Supplementary Figure S3:** PRISMA Flowchart of literature review and search strategy for studies reviewed for **Unmet Need 4: Management of stricturing Crohn's disease.** Search Terms: *((((Crohn's Disease[Mesh Terms]) AND (Stricture)) OR ((Crohn's Disease[Mesh Terms]) AND (Stricture Management))) OR ((Crohn's Disease[Mesh Terms]) AND (Endoscopic Balloon Dilatation))) OR ((Crohn's Disease[Mesh Terms]) AND (Strictureplasty))* Results included studies from 2013-2023.

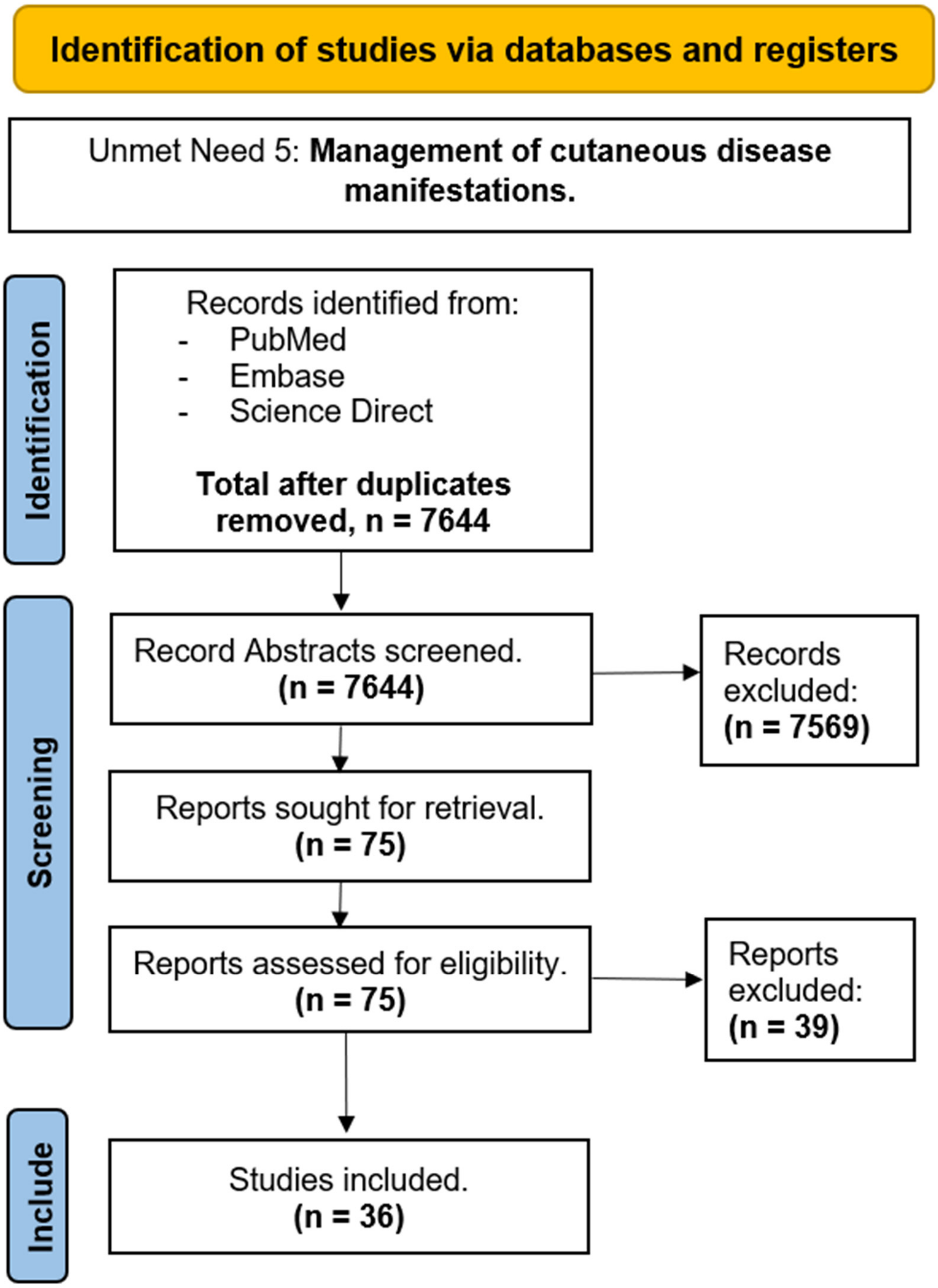


**Supplementary Figure S4:** PRISMA Flowchart of literature review and search strategy for studies reviewed for **Unmet Need 5: Management of cutaneous disease manifestations.** Search Terms: *(((((Contiguous Crohn's Disease) OR ((Cutaneous Crohn's Disease) AND (Treatment))) OR ((Cutaneous Crohn's Disease) AND (Biologics))) OR (Metastatic Crohn's Disease)) OR ((Crohn's Disease) AND (Dermatological Manifestations))) OR ((Crohn's Disease) AND (Cutaneous Manifestations))* Results included studies from 2013-2023.
